# A unified framework for developing effective hygiene procedures for hands, environmental surfaces and laundry in healthcare, domestic, food handling and other settings

**DOI:** 10.3205/dgkh000293

**Published:** 2017-06-19

**Authors:** Sally F. Bloomfield, Philip C. Carling, Martin Exner

**Affiliations:** 1 London School of Hygiene and Tropical Medicine, London, United Kingdom; 2 International Scientific Forum on Home Hygiene, Montacute, Somerset, United Kingdom; 3 Department of Infectious Diseases, Carney Hospital and Boston University School of Medicine, Boston, USA; 4 Institute of Hygiene and Public Health, University of Bonn, Bonn, Germany

**Keywords:** infection prevention, hygiene, cleaning, disinfection, biocide, hands, laundry, environmental surfaces

## Abstract

Hygiene procedures for hands, surfaces and fabrics are central to preventing spread of infection in settings including healthcare, food production, catering, agriculture, public settings, and home and everyday life. They are used in situations including hand hygiene, clinical procedures, decontamination of environmental surfaces, respiratory hygiene, food handling, laundry hygiene, toilet hygiene and so on.

Although the principles are common to all, approaches currently used in different settings are inconsistent. A concern is the use of inconsistent terminology which is misleading, especially to people we need to communicate with such as the public or cleaning professionals.

This paper reviews the data on current approaches, alongside new insights to developing hygiene procedures. Using this data, we propose a more scientifically-grounded framework for developing procedures that maximize protection against infection, based on consistent principles and terminology, and applicable across all settings.

A key feature is use of test models which assess the state of surfaces after treatment rather than product performance alone. This allows procedures that rely on removal of microbes to be compared with those employing chemical or thermal inactivation. This makes it possible to ensure that a consistent “safety target level” is achieved regardless of the type of procedure used, and allows us deliver maximum health benefit whilst ensuring prudent usage of antimicrobial agents, detergents, water and energy.

## Zusammenfassung

Hygienemaßnahmen für Hände, Oberflächen und Textilien sind entscheidend zur Verhütung der Weiterverbreitung von Infektionserregern in unterschiedlichen Bereichen wie medizinischen Einrichtungen, in der Lebensmittelproduktion, Gastronomie, Landwirtschaft, öffentlichen Einrichtungen, im häuslichen Umfeld und im täglichen Leben. Sie werden angewendet in Situationen wie der Händehygiene, bei Durchführung medizinischer Eingriffe, zur Desinfektion und Reinigung von Flächen, beim Husten und Niesen, beim Umgang mit Lebensmitteln, bei der Wäschehygiene und bei der Toilettenhygiene etc.

Obwohl die Prinzipien überall die gleichen sind, sind die Herangehensweisen in den verschiedenen Bereichen unterschiedlich. Sorge bereitet die unterschiedliche Terminologie, die missverständlich ist, insbesondere gegenüber Personen, mit denen hierbei kommuniziert werden muss wie der Öffentlichkeit und dem Reinigungspersonal.

Der Artikel stellt die Daten für die unterschiedlichen Konzepte und die neuen Entwicklungen zur Entwicklung von Hygienestrategien dar. Unter Berücksichtigung dieser Aspekte empfehlen wir eine mehr wissenschaftlich-basierte Strukturierung für die Entwicklung von Strategien, die den Schutz vor Infektionen wirksamer werden lässt, basierend auf konsistenten Prinzipien und einer einheitlichen Terminologie und Anwendung für alle Bereiche.

Ein Schlüsselelement ist die Anwendung von Testmodellen, die den Status einer Oberfläche nach Behandlung prüft und nicht nur die Leistung des Produkts. Das erlaubt es Produkte, die die Entfernung von Mikroorganismen überprüfen, zu vergleichen mit solchen Produkten, die auf einer thermischen oder chemischen Inaktivierung basieren. Das ermöglicht es sicherzustellen, dass unabhängig von der Art der Anwendung ein konsistenter Sicherheitsgrad erreicht wird, und erlaubt gleichzeitig die Erzielung eines maximalen gesundheitlichen Nutzeffekts bei richtiger Anwendung antimikrobieller Agentien, Detergentien, Wasser und Energie.

## Introduction

Hygiene procedures applied to hands, surfaces and fabrics are central to preventing spread of infection in a whole range of settings including healthcare, food production, catering, agriculture, public and home and everyday life settings. It is used in a whole variety of hygiene situations including hands, clinical procedures, decontamination of environmental surfaces, respiratory hygiene, food handling, laundry hygiene, toilet hygiene and so on. 

An essential fact that is sometimes overlooked is that the basic principles of infection transmission and its prevention are the same regardless of the setting or situation:

- Regardless of setting (hospital, domestic, public, food handling etc.), infection transmission is based on the principle that pathogenic bacteria, viruses and fungi are constantly shed from a range of primary sources including infected and colonized people, raw food, water and animals, and circulate around these settings so that, when circumstances combine, people become exposed, colonized and infected. Infection prevention is about applying hygiene procedures appropriately to break the chain of infection transmission.
- Regardless of the type of surface (hands, environmental surfaces, fabrics) the object of a hygiene procedure is to reduce contamination to an acceptable safety target level. This is achieved by applying a procedure which removes the pathogens from the surface, using cleaning products such as detergents or hand soap with running water, or by applying a product or process that inactivates the microbes *in situ* (heat, UV radiation, disinfectants). Most usually it involves a combined procedure of removal and inactivation, applied sequentially or in combination.

Despite the fact that these principles apply in all settings, attitudes and approaches, having developed from different historical roots, differ considerably. In clinical settings, hands, high frequency touch surface and laundry hygiene procedures are assessed separately, and the need for a hygiene procedure established for each situation through intervention studies which assess the impact on infection rates. In food processing and domestic hygiene, on the other hand, a multibarrier approach based on risk assessment is used in which critical control points requiring hygiene intervention are identified from microbiological data. Although different, these approaches are not conflicting, and increasingly, risk assessment approaches are being used in healthcare settings.

By contrast, although the principles applied to hygiene procedures themselves are common to all settings, the approaches currently used in different settings in developing, assessing and communicating hygiene procedures are inconsistent and sometimes conflicting. Presently there is a tendency to consider hand hygiene separately from other procedures. Detergent-based cleaning of hands is seen as a means to reduce contamination to a level considered as safe, but detergent-based cleaning of environmental surfaces, particularly in healthcare settings, is regarded as the means to remove soil prior to application of a disinfectant; the fact that, as with hand hygiene, cleaning itself contributes to achieving safety target levels on surface is ignored. The efficacy of hygiene procedures involving removal (e.g. detergent-based cleaning) is rarely compared with those involving inactivation (e.g. disinfectant use) despite the fact that both are intended to produce the same result. If hygiene procedures are to deliver real health benefits, we need a consistent approach based on scientific principles, which can be applied across all settings.

A particular concern is the inconsistent terminology which we use, which is misleading, especially to the people we particularly need to communicate with such as the public or those with responsibility for implementing hygiene measures who may have limited training and little technical understanding. Regardless of which setting we work in, we also share responsibility for preventing spread of infection within our family, household and everyday life, and much of our understanding about hygiene originates from childhood. If we are to contain the overall burden of infection in our lives, and address the problem of antibiotic resistance, hygiene must be the shared responsibility of everyone, regardless of whether we are infection control professionals or not. This will not happen unless we adopt consistent approaches and terminology through which we can communicate with each other – regardless of where we live and work, and our level of education and training in infection transmission.

In this paper we review current approaches, alongside new insights and new approaches, to developing effective hygiene procedures to break the chain of infection transmission. These are used to propose a more rational and scientifically-grounded framework for developing procedures (products and processes) that maximize protection against infection exposure, based on consistent principles and terminology, and applicable across all settings where infection prevention and control (IPC) is important. The discussion will be confined to those sites and surfaces which interact in an interdependent manner to spread infections around occupied settings, It will not include procedures for processing critical surfaces such as medical instruments used in healthcare situations.

## The need for unifying terminology

A particular problem is the lack of an agreed definition for the process of “reducing the numbers of pathogens on surfaces to an acceptable safety target level”, and the state (assurance that pathogens have been reduced to a safe level) of the surface following the procedure. In practice the terms “cleaning” and “clean” are commonly used to describe this process and state, regardless of whether it involves mechanical removal and/or disinfection of pathogens. The problem arises because this term “cleaning” is also used to describe that part of the process which involves mechanical removal of visible soil and contamination.

Consistent terminology is not only important at scientific level, it is vitally important for communicating with healthcare workers and the public who are responsible for such activities. For most people “clean” means absence of visible dirt. Advising consumers (or cleaning staff) to "clean" a surface means that they will clean until the visible dirt is gone. Increasingly the data [1] show that potentially unsafe levels of pathogens can remain on visibly clean surfaces. It needs to be made clear that, in hygiene practice, a surface can only be judged to be “safe” if it has been subjected to a validated hygiene procedure which has been carried out in the prescribed manner. Visible cleanliness alone is not sufficient to judge whether a surface is safe.

In order to avoid confusion and errors in hygiene practice, there is need for an agreed definition which clearly implies a process or state where the number of pathogens is reduced to a safe level. Unqualified use of the term “cleaning” should be avoided in scientific writing and in communicating hygiene practices with cleaning staff, the public etc. Terms other than cleaning which could be used to define this process and state include hygienic cleaning (hygienically clean), decontamination (decontaminated), cleaning and disinfection (clean and disinfected), dis-infection (dis-infected) or sanitization (sanitized). For consistency in this review, we have used the term “hygienic cleaning” to refer to any process intended to reduce the numbers of pathogens on surfaces to an acceptable safety target level which makes it fit for purpose, and “hygienically clean” to define the state of that surface after hygienic cleaning.

## Developing an integrated approach to optimizing hygienic cleaning procedures

If hygiene procedures are to be effective, we need to determine not only product efficacy (proof of principle), but more importantly, whether the procedure results in what we want to achieve – namely hands, environmental surfaces, fabrics etc. which are hygienically clean (i.e. fit for purpose), as sufficient to break the chain of infection transmission. An approach which focuses on establishing “fitness for purpose” of a hygiene procedure, must take account of three things:

- The combined effectiveness of removal and inactivation processes when used together or sequentially.
- The effectiveness of the procedure in preventing onwards transmission of contamination
- The time period over which the safety target level can be, or needs to be, sustained

These 3 aspects are considered in the following sections

### A hygiene assurance framework for quantifying and comparing hygiene procedures involving removal and inactivation

**Figure 1:**
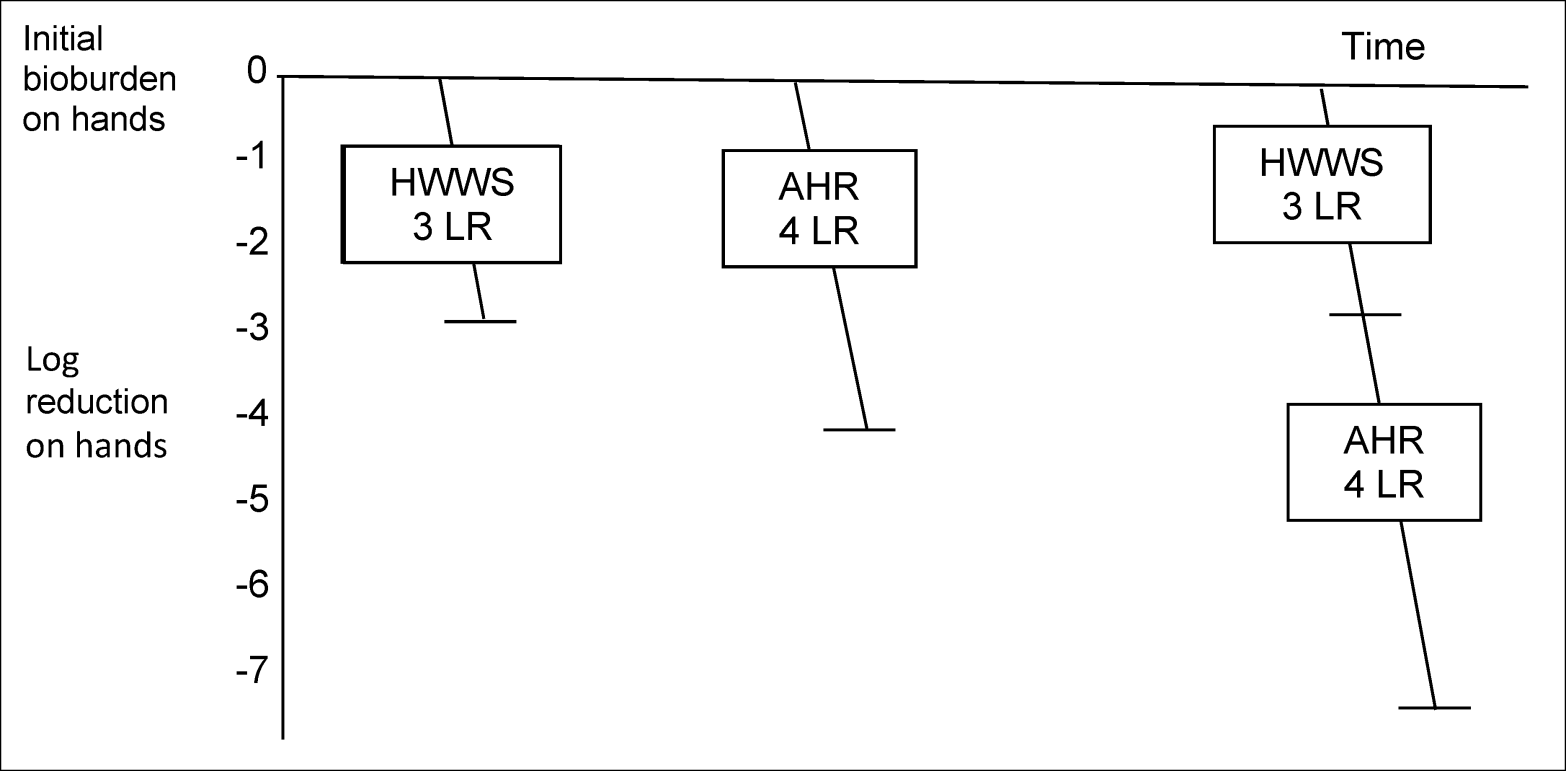
Hypothetical visualization of Log_10_ reduction (LR) of contamination on hands following handwashing with soap (HWWS), use of alcohol hand rub (AHR), and HWWS followed by use of AHR

**Figure 2:**
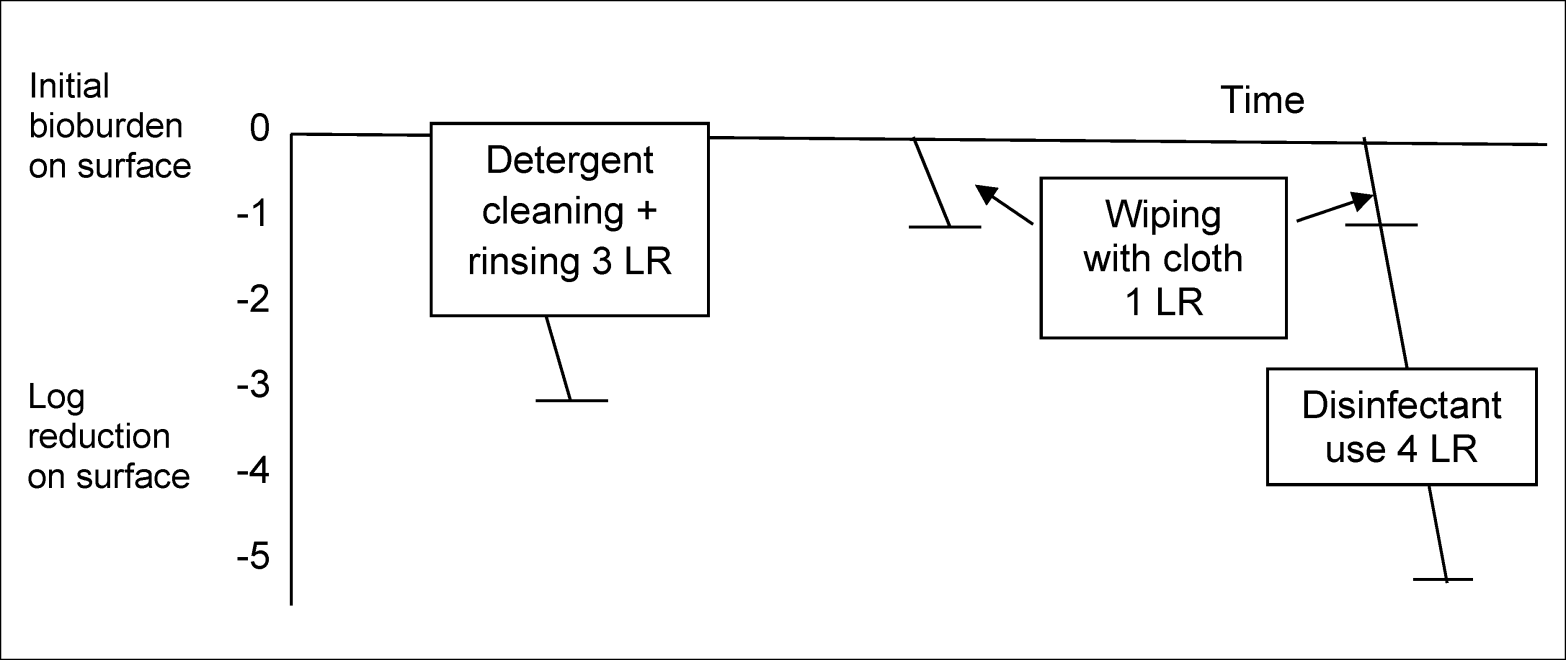
Hypothetical visualization of Log_10_ reduction (LR) of contamination on environmental surfaces following detergent-based cleaning with rinsing, wiping, and wiping followed by disinfection

**Figure 3:**
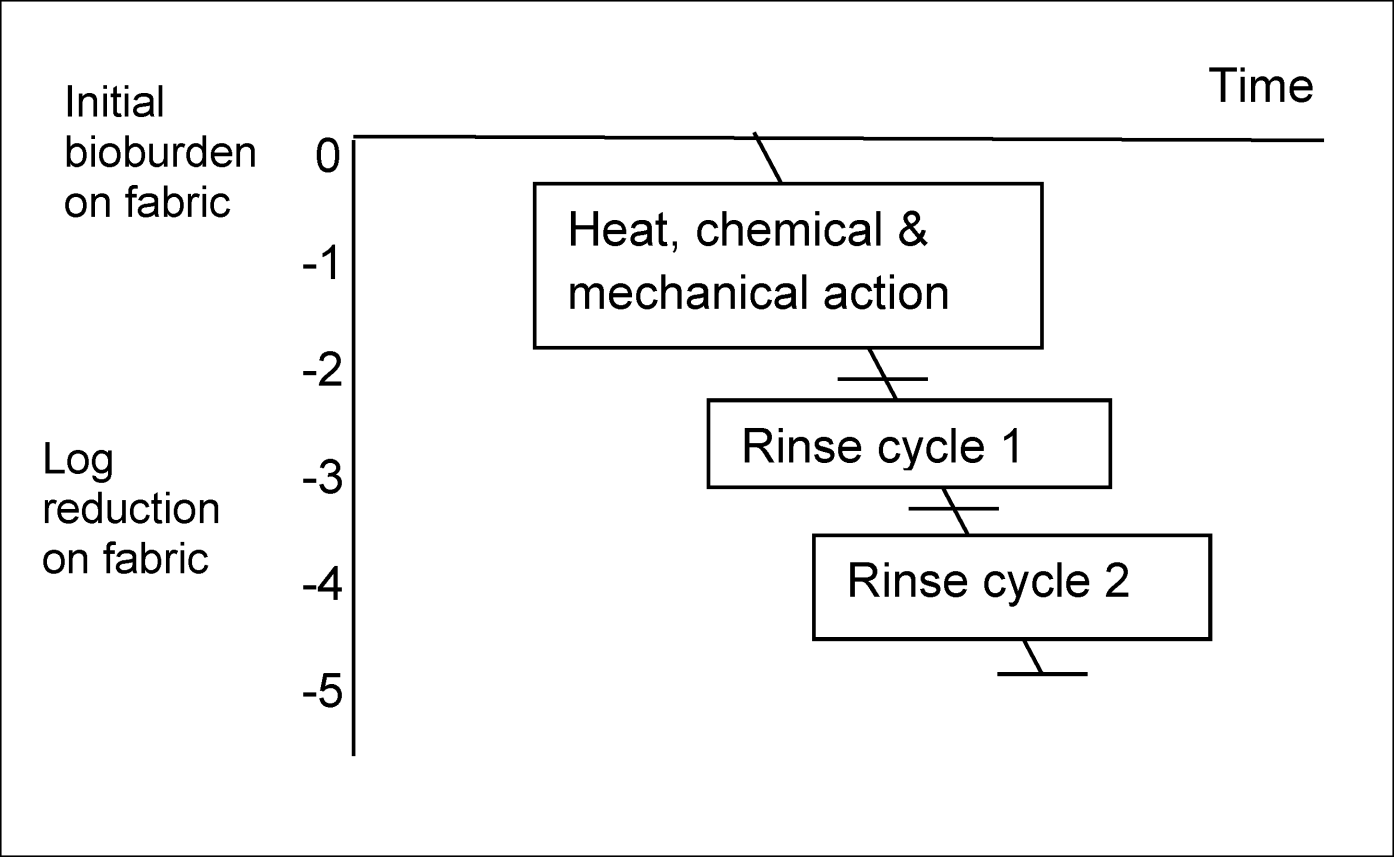
Hypothetical visualization of Log_10_ reduction of contamination on fabrics during machine laundering

In this section we propose a “hygiene assurance” framework which could be used for quantifying and comparing the efficacy of hygiene procedures (and ensuring their equivalence) regardless of whether they involve removal by cleaning or inactivation with disinfectant, or a combination of both. Figure 1 (Fig. 1), Figure 2 (Fig. 2), and Figure 3 (Fig. 3) illustrate how this might work, although many of the values used in these figures are hypothetical, because requisite data is not currently available. 

#### Hand hygiene procedures

For hands, handwashing with soap (HWWS) is used as a means to produce hands which are hygienically clean, provided the specified technique is used [2]. Data suggests that HWWS, if carried out as specified, can produce 0.5 up to 3 or more Log_10_ reduction (LR) in bacterial contamination on hands [3], [4] (Figure 1 (Fig. 1)) If soap and water are unavailable, other processes are needed which achieve an equivalent LR. Data suggests that alcohol hand rubs (AHR) (also referred to as alcohol hand sanitizers) are acceptable, which (apart from some non-enveloped viruses e.g. hepatitis A) produce equivalent or greater than 3 LR on hands [5]. In higher risk situations where the initial bioburden on hands may be higher or the safety target level may be lower (e.g. before changing dressings or catheter care, or after changing a nappy or handling raw chicken) it may be advisable to recommend HWWS followed by use of AHR. The data suggests that working in sequence, this could produce an LR of up to 7.

#### Environmental surface hygiene procedures

For hand touch or food contact surfaces, (referred to as “critical control points” in the food etc. industries, and “high frequency touch surfaces” in healthcare settings), again there is remarkably little data available on the efficacy of hygiene procedures involving dry wiping or detergent-based cleaning, despite the fact that they are used as a means to achieve a hygienically clean surface. In the last few years some limited number of studies have been reported as discussed in a later section below. 

Where a surface is dry wiped or wiped with detergent solution using a cloth etc., the log reduction on the surface will be the difference between the amount of soil and microbes detached from the surface onto the cloth and those re-deposited from the cloth to the surface. As illustrated in Figure 2 (Fig. 2), it is reasonable to expect that the log reduction will be less than that resulting from detergent-based cleaning followed by rinsing under clean running water. Whereas a 1 log reduction by wiping alone might be considered sufficient for hygienic cleaning of low risk surfaces such as floors and furniture, it may be that, for critical control points/high frequency touch surfaces which cannot be rinsed, such as food preparation surfaces, hospital bedrails, toilet seats and flush handles, door handles, etc., additional use of a disinfectant may be needed to ensure that an LR value equivalent to that produced by detergent cleaning with rinsing is achieved. For higher risk situations (high surface bioburdens after preparing a contaminated chicken or where there are persons with impaired immunity) it may be concluded that combined cleaning and disinfection which produces greater than 3 LR is needed.

#### Fabric laundering procedures

For clothing and bed linens etc., hygienic cleaning is achieved by combined action of heat inactivation and removal during machine wash and rinse cycles. Efficacy can be enhanced by using active oxygen bleach (AOB)-containing detergents, which release active oxygen which contributes some microbicidal action [6]. Figure 3 (Fig. 3) shows a hypothetical representation of the components of a laundry cycle. 

At present, many experts advocate that the decision to use a disinfectant should be based on the level of risk i.e. they should only be used in healthcare situations where there is an infected person, or a person with compromised immunity [7]. Conversely, some expert bodies such as the Centres for Disease Control (CDC) recommend the use of hospital grade disinfectants on all patient zone surfaces [8]. The hygiene assurance framework proposes that we should start by deciding the safety target level considered to be appropriate for the situation, and then consider whether this can be achieved by cleaning alone, or whether a disinfectant is needed to reach the target level. This means, for example, that even in normal situations, although detergent-based cleaning with rinsing surfaces (including hands, frequent touch, or food contact surfaces) may be sufficient, in situations where rinsing is not possible (no access to running water, or surfaces that cannot be rinsed) disinfection or AHR-based interventions may be needed to achieve the equivalent safety target level.

Once established, using a “hygiene assurance” approach allows us to address a number of issues, namely to:

- compare the hygiene efficacy of dry wiping or detergent-based cleaning and rinsing, with the efficacy of cleaning combined with disinfection.
- determine the total effect of combined inactivation/removal processes such as laundering, and quantify the separate contributions of each component
- ensure that new hygiene procedures (product and process) are at least as effective as existing ones.
- apply the same basic principles to hygienic cleaning of all types of surfaces including hands, environmental surfaces, laundry, dishwash in all types of settings
- work to different “safety target levels” according to level of risk to the patient or household member, and the size of the initial bioburden
- demonstrate in a quantitative manner how compliance with the recommended method of application (handwashing technique, disinfectant contact time etc.) is equally as important as the product used.

Although a hygiene assurance level approach could be very useful for comparing hygiene efficacy across a range of situations, it is important that it is not used inappropriately to set performance requirements unless or until more comprehensive data is available on the relationship between LR and infection risk reduction. This is further discussed below.

### Testing the effectiveness of hygiene procedures 

Based on the principles outlined above, Table 1 (Tab. 1) proposes a 4 stage hierarchy of testing for establishing efficacy of hand, surface and laundry hygiene procedures.

Testing performance through stage 1 and stage 2 suspension and surface tests provides proof of principle for procedures involving disinfectants and AHRs. These methods can also be used to assess, for example, the separate LR contribution made by active oxygen bleach (AOB) in a laundry detergent [9]. Surfactants in soap and detergents can themselves contribute some microbicidal (bactericidal, fungicidal, virucidal etc.) action [9] although there are few quantitative data; in a study, using suspension tests, Kim et al. [4] evaluated the bactericidal activity of plain hand soap against 20 bacterial strains for 20 sec at 22°C. LR values for the different strains varied from zero up to as much as 1.5. 

The purpose of stage 3 is to show whether a hygiene procedure (detergent-based, wiping, cleaning and disinfection etc.) delivers fitness for purpose i.e. delivers a specified safety target level on hands, surfaces and fabrics under controlled conditions simulating usage. It is also used to determine whether the procedure prevents onward transmission of microbes. 

Stage 1, 2 and 3 test methods are further reviewed by Gebel et al. [10]. 

The purpose of stage 4 is to provide quantitative data linking effectiveness of procedures to reductions in infection risk. This aspect is discussed later.

### Establishing the effectiveness of hygiene procedures – developing test models

As stated above, bactericidal, fungicidal or virucidal activity of disinfectants (hand, surface and laundry) is initially established (stage 1) by suspension tests which demonstrate the ability of the product to produce a given LR (usually 3-5 LR) using test strains and conditions related to intended use. Efficacy of hard surface disinfectants is further established by stage 2 tests in which inoculated surface carriers are exposed to products at use dilution [11], [12]. These tests are important, since comparative studies of disinfectant products [13], [14] show that, in general, efficacy against surface dried films is significantly lower than against organisms in suspension. 

Establishing fitness for purpose of a hygiene procedure through stage 3 models involves testing under conditions which are as close as possible to conditions of use. Stage 3 should also demonstrate that the procedure is sufficient to prevent onward transmission of pathogens. Developing stage 3 models represents a challenge. A major difficulty is ensuring repeatability and reproducibility. The closer the conditions are to practice the more difficult it becomes to control variables [15]. To an extent, this can be optimized by using standard test methods for preparing test inocula, and pre and post enumeration of organisms, but in some cases entirely new methods are needed e.g. for testing of wipes.

Stage 3 models also need to take account of the fact that microbes tend to attach to wet surfaces and form resident biofilms, which can become detached and transferred to dry surfaces where they can survive for extended periods. Biofilms are generally less susceptible to disinfectants and more resistant to physical removal [16]. A study by Stewart et al. [17] describes rebound of *Staphylococcus aureus* and methicillin-resistant *S. aureus* (MRSA) levels on hospital surfaces 24 h after cleaning and disinfection. It was suggested that this might be due to removal of biofilm by the disinfectant allowing release of planktonic staphylococci from microscopic crevices on the surfaces, although no investigations were carried out to substantiate this.

In recent years, numerous laboratory and field studies have been published which are increasing our understanding of how pathogens are shed from infected sources and transmitted via hand, environmental and fabric surfaces [18]. These offer methodology for developing stage 3 test models appropriate to different use conditions. Some examples are outlined in the following 3 sections.

#### Hand hygiene procedures

Stage 3 efficacy of hand hygiene procedures is established by panel test models, many of which have been developed as standard tests [2], [19], [20], [21]. These include tests developed by the Comité Européen de Normalisation (CEN tests) and the American Society for Testing Materials (ASTM tests). These test methods determine LRs on artificially contaminated hands. Standard handwashing panel tests are stage 3 tests since they assess efficacy of removal as well as inactivation. Data from studies of the efficacy of hand hygiene procedures is summarized in Table 2 (Tab. 2).

Although HWWS is probably the most important personal hygiene intervention, there is surprisingly little data on efficacy of handwashing with plain soap (i.e. soap which does not contain any microbicidal agents), most particularly for viruses and fungi. Data from a ring trial of EN 1499 handwash test carried out in 15 laboratories (presented in the annex to the standard) [2] indicate that the mean LR of *Escherichia coli* on contaminated hands after a 30 s handwash with plain soap (soft soap is used in this test) is 2.76 (range 2.02–4.27). Kim et al. [4] found that washing with plain soap for 30 sec according to a prescribed routine produced a 1.96 LR in *Serratia marcescens* inoculated onto hands. Steinman et al. [22] showed that 5 min handwashing with plain soap produced >2 LR of poliovirus inoculated onto hands, compared with only a 1 LR where volunteers were asked to wash their hands using “daily routine” procedure (10–55 sec). Ansari et al. [19] showed that HWWS for 10 sec produced a 72.5% reduction (0.56 LR) in rotavirus on hands. Schürmann and Eggers [23] found that 30 sec handwashing with soap produced an LR reduction of 1.9.

Although, where there is no access to running water, AHR is used as an alternative to HWWS, efficacy of AHRs relative to HWWS is rarely compared. Infection control guidelines often advise against using AHRs where norovirus, rotavirus, rhinovirus etc. is suspected, considering them as ineffective against viruses with no lipid envelope, despite the fact that this has not been verified by intervention studies. Set against this, although the summary of data (Table 2 (Tab. 2)) from different studies of HWWS and AHR use (at concentrations used in the domestic rather than hospital settings) confirms that the efficacy of AHR against non-enveloped viruses, is generally less than against enveloped viruses and bacteria when tested using stage 1 suspension tests, it suggests that (with the exception of hepatitis A virus) efficacy of AHR against non-enveloped viruses is no less than that of HWWS against bacteria when tested using a stage 3 panel test model. Since only 2 studies on efficacy of HWWS against viruses were identified, it is difficult to say whether this also applies to HWWS against viruses. However, in a 2015 fingerpad study, Tuladhar et al. [24] found that murine norovirus infectivity reduction by HWWS for 30 sec (>3.0 log_10_) was significantly higher than treating hands with AHR (propan-2-ol 45% (w/w), propan-1-ol 30% (w/w) which produced 2.8 LR. By contrast Paulmann et al. [25], using the ASTM fingerpad method [26], found that 70% ethanol produced a 4.69 LR in murine norovirus on hands compared with only 2.86 when hands were treated with water and rinsed.

#### Environmental surface hygiene procedures

Stage 3 models are increasingly being used to assess inactivation by disinfectants, but are still rarely used to evaluate removal of contamination from environmental surfaces by detergent-based cleaning or dry wiping procedures. Equally important they are rarely used to compare efficacy of hygiene procedures involving cleaning with those involving cleaning and disinfection. 

Table 3 (Tab. 3) summarizes LR values from studies of procedures based solely on removing contamination from surfaces, including dry wiping and detergent-based wiping with or without rinsing. These suggest values from 0.2 up to 3.6 LR, with most values lying between 1.5 and 2.5. Although these data are useful in suggesting the order of magnitude of reductions in contamination produced by cleaning alone, they give little indication of the relative efficacy of the different approaches (e.g dry wiping versus wet wiping), since most (all but three) studies evaluated only one approach. The variability in data obtained from different studies as illustrated by Table 3 (Tab. 3), likely due to variations in test conditions, precluded any meaningful comparisons across studies. In two studies [27], [28], investigators were able to demonstrate that LR values achieved by dry wiping with microfibre cloths, particularly ultramicrofibre cloths are superior to conventional cloths, but none of the studies compared the effectiveness of dry wiping with detergent-based wiping. In a study [29] where detergent-based cleaning and rinsing was compared with detergent-based wiping alone, surprisingly this showed only limited increase in effectiveness where wiping with detergent was followed by rinsing. This is in contrast to the studies of Cogan et al. [30] where participants prepared meals using chickens contaminated with *Salmonella* spp. or *Campylobacter* spp. These data showed that, during preparation, contamination was spread to hands, hand and food contact surfaces, and cleaning cloths, with 17.3% of surfaces showing contamination. Where participants cleaned up by wiping with a cloth soaked in detergent and hot water, there was no significant reduction in contamination. By contrast, a follow-up study, using the same methodology [31] showed that, after wiping alone, 3.3% and 33% respectively of sites sampled (hands, hand and food contact surfaces, and cleaning cloths) showed *Salmonella* and *Campylobacter* counts of >1000 colony forming units (cfu). By contrast, wiping cleaning with detergent and a cloth followed by rinsing under running water (10 sec) reduced campylobacter-contaminated sites to 1.7% with no sites showing greater than 100 cfu. For *Salmonella*, 16.7% of sites still showed contamination, with 3.3% of sites showing counts >100 cfu. The study was used to develop a stage 3 test for comparing procedures for reducing cross contamination during food preparation [32].

A whole range of studies carried out using stage 3 laboratory or field models are reported, which mostly demonstrate that cleaning and disinfection is more effective than detergent-based cleaning alone [30], [31], [32], [33], [34], [35], [36], [37], [38], [39]. However, none of these stage 3 model studies were carried out in a manner which allowed the separate LR contributions of cleaning (removal) and disinfection (inactivation) to be quantified i.e. compared the LR achieved by cleaning alone with that achieved by cleaning and disinfection. The literature also contains a range of field studies evaluating the efficacy of cleaning and disinfection in hospital environments (as reviewed by Dancer [7], [40]) but only some, compare the efficacy of different approaches in order to determine which might be the most effective.

One area of application which has prompted development of test methods which measure combined removal and inactivation is the increasing use of detergent and disinfectant wipes for high frequency touch surfaces. Sattar and Maillard [41] critically reviewed data from studies using various methods, but few methods included controls to allow assessment of the added value of using a disinfectant wipe over a detergent wipe. In a 2015 study, Sattar et al. [42] assessed the new ASTM standard [43] for evaluating detergent and disinfectant wipes. In this test, wiping is carried out using a purpose built wiperator to deliver a standard orbital motion for 10 sec at a pressure of 150 g. All of 5 disinfectant pre-soaked wipes tested achieved a LR >4 of *S. aureus* and *Acinetobacter baumanii*, but only one (containing 0.5% accelerated H_2_O_2_) prevented transfer of bacteria to another surface. A control wipe was included which produced around 3 LR, but this was soaked in buffer rather than detergent.

The need to distinguish visibly from hygienically clean is demonstrated by Carling et al. [44]. In this study, 12 near patient surfaces (bed rail, call button, telephone, tray table, bathroom door, sink, and grab bar, toilet handle and seat) were sampled. Surface cleanliness was monitored by treating portions of the surface with fluorescent marker, whilst total counts were confirmed by dip slides. By simultaneously evaluating disinfection and cleaning, it was possible to analyze components independently (process and product). For disinfectant A and B, 40% of 237 and 77% of 274 surfaces respectively, confirmed as clean by fluorescent marker removal, were found to have complete removal of aerobic bioburden. Because there was no difference in thoroughness of cleaning with either disinfectant (65.3% and 66.4%), the difference in bioburden reduction can be attributed exclusively to better hygienic cleaning efficacy with disinfectant B. 

#### Laundry hygiene procedures

Standard methods such as those developed by International Electrotechnical Commission (IEC), CEN and ASTM or adaptations of these tests are used to evaluate laundry disinfectants [45], [46], [47]. These stage 3 models measure efficacy (microbial removal plus inactivation) of machine wash and rinse cycles using artificially contaminated fabrics. 

A review of 25 published studies of the hygiene efficacy of machine laundering [6], indicated that, although there was significant variability between data from different studies due to lack of standardization of test conditions, machine laundering can produce up to 3–6 or more LR depending on temperature, detergent formulation, wash cycle time, number of rinse cycle etc. Data suggest that rinse cycles probably contribute around 1 LR (each) [6], whilst use of AOB-containing detergents can increase the LR value by 1 or more, depending on temperature and test strain [48]. Brands et al. have also carried out stage 2 suspension test methods to assess the extent to which inactivation by heat over the temperature range 20–60°C, with or without the addition of AOB contribute to the overall reduction in contamination during machine laundering [9]. 

## Preventing onwards transmission of contamination

Since the primary aim of hygienic cleaning is to break the chain of infection transmission, in addition to measuring the LR in contamination on surfaces, the efficacy of the procedure in preventing onward transmission of contamination should also be assessed as an integral part of the evaluation process. It must also be borne in mind that solutions used for mopping and rinsing, and the mops, cloths or detergent wipes, will becomes increasingly contaminated during hygienic cleaning, and can serve as a medium for spreading microbes around the environment.

A number of studies are reported, both laboratory and field studies, which demonstrate that, if significant contamination remains on surfaces after cleaning, or cleaning and disinfection, this is readily spread to other surfaces via hands and cleaning utensils [30], [31], [35], [38], [41], [49], [50], [51], [52], [53], [54]. Bloomfield et al. [6] review studies showing that, where machine laundry wash cycles are inadequate, transfer occurs from contaminated to sterile items included in the wash cycle. 

Exner et al. [35] and Williams et al. [50] describe stage 3 models which have been specifically designed to assess onward transmission of pathogens from treated surfaces. These are further reviewed by Gebel et al. [10]. The test model; developed by Exner et al has now been published as a European standard [55].

## Setting performance requirements for hygienic cleaning procedures

While it is accepted that the purpose of a hygiene intervention is to reduce numbers of pathogens on surfaces to an acceptable safety target level, we currently lack a science-based framework for defining such a condition [56].

To set performance requirements for hygiene procedures, data are needed on the levels (bioburdens) of potentially harmful and/or antibiotic resistant strains found on sites and surfaces. For surfaces which are cleaned and/or disinfected at intervals, such as floors etc., and for frequent touch surfaces such as touch plates, door handles, wash taps, toilet flush handles etc., we need to know typical ambient bioburdens. For procedures which involve intervention at specified times, we need to know that typical bioburden at the critical time when intervention is needed, which will be quite from the ambient bioburden. For example, it is important to know the bioburden which might be present on hand contact surfaces where a patient or family member is a shedder of pathogenic or resistant strains, or on hand and food contact surfaces which have recently been used for preparation of contaminated poultry. Although data on types of pathogens likely to be found on critical surfaces is increasing, data on bioburden levels at critical times is limited [18].

We also need to decide on the safety target level i.e. a level of residual contamination for which the infection or colonization risk is also reduced to a level appropriate to the situation. This is difficult since the “infectious dose” of bacteria and viruses varies considerably for different organisms and different situations [18], [57]. The “infectious dose” for common pathogens such as *Campylobacter* or enterohemorrhagic *E. coli* (EHEC) and *Clostridium difficile*, but particularly for viruses such as norovirus and rhinovirus, can be very small (1–500 particles or cells) [18], [57]. For others e.g. the oral dose of *Salmonella* spp., it can be much higher (up to 10^6^ organisms) [18], [57]. The infectious dose also depends on host susceptibility and may be lower for “at risk groups” with compromised immunity. 

Three approaches offer the possibility for setting performance requirements for hygienic cleaning processes.

### Pragmatic approach

Presently, the most-used approach is a pragmatic approach, where performance criteria are based on LRs which we could reasonably expect to achieve. The precedent for this is seen in CEN and EPA standard disinfectant test methods where “pass” levels of 3, 4 to 5 LR are set for suspension and surface tests. These requirements, which we have accepted for 20 or more years, are not based on any clinical knowledge (i.e. intervention study data showing reduction in infection rates), but on the basis that they are known to be achievable by currently-used disinfectants. By contrast, acceptable “pass” levels and requirements are rarely considered for procedures which involve removal of pathogens such as HWWS; the effectiveness of HWWS in preventing transmission of infection is mostly assumed. 

### Clinical intervention studies

Carefully designed clinical intervention studies have recently demonstrated the impact of interventions on infection rates and patient acquisition rates in healthcare settings. These studies are reviewed by Donskey [58], Carling and Huang [1] and Dancer [7], [40], and include interventions measuring the impact of terminal disinfection of hospital rooms and use of copper impregnated high touch surfaces in hospital settings. Hand hygiene intervention studies have also been used to estimate reductions in gastrointestinal and respiratory infection rates in community settings [3]. Although some studies in healthcare settings show a causal relationship between surface bioburden and infection risk [59], using such data to set performance standards is not a feasible option, requiring extensive dose-response intervention studies to quantify the decrease in surface bioburden required to produce the incremental decrease in infection transmission risk deemed appropriate to the situation. 

We also need to decide on the safety target level i.e. levels of residual contamination after hygienic cleaning which no longer constitute an infection or colonization risk. This is difficult since the “infectious dose” of bacteria and viruses varies considerably for different organisms

Aiello and Larson [60] point out that, although a single control point, such as the hands, may be a “sufficient cause”, infection transmission usually involves a number of interdependent “component causes” such as the hands, touch surfaces, food contact surfaces, cleaning cloths and utensils, laundry, baths, basins, air etc. which act together to determine the overall risk. Thus, although this approach may be applicable to interventions such as hand hygiene, this is not so for other sites and surfaces. Whilst intervention studies for non-critical environmental surfaces are feasible in hospital situations where infection rates are higher, this is not so for public or domestic hygiene where very large test populations would be required to obtain significant results.

### Quantitative Microbial Risk Assessment

In the last 20 years Quantitative Microbial Risk Assessment (QMRA) has been increasingly used to estimate the relationship between the LR in contamination produced by a hygiene procedure and reduction of infectious disease risk [61], [62], [63], [64]. The study by Ryan et al. [64] illustrates how QMRA could also be used to set safety target levels i.e. for estimating the LR on a surface needed to reduce the infection risk to an acceptable level. 

For each of 7 microorganisms, data on infectious dose, surface occurrence, transfer efficiency, exposure assessment etc. were extracted from the literature and infection risk determined for a scenario where a contaminated surface was touched with the fingers, and the fingers then touched the mouth, nose or eyes. A target of a 1 in 1 million (10^–6^) risk of infection per touch was set as the safety target level (deemed the acceptable daily exposure risk for drinking water [64]). Using dose-response models obtained from the QMRA website [65], hand to mouth infection risk estimates ranged from 10^–3^ for norovirus down to 10^–9^ for staphylococci for a single touch of the contaminated surface. Further analysis suggested that, on average, 2 LR was sufficient to achieve the 10^–6^ safety target level for *E. coli* and *Listeria* spp., whilst norovirus required an LR of 3.44. For *Pseudomonas* spp., *Salmonella* spp., and *S. aureus* it was estimated that no decontamination process was required.

Interestingly, a comparison of the LR values calculated as sufficient to mitigate infection risk, with proposed EPA standards showed was that the computed values were generally lower than EPA requirements. For example, the EPA proposal of 99.9% for “non food contact surface sanitizers” is higher than the 99% reduction determined by QMRA. However the estimates were based on literature data for ambient concentrations of bacteria and viruses on surfaces, mostly taken from domestic and public settings. Whereas the computed values may be appropriate for routine cleaning of environmental surfaces, they are inappropriate for critical control points/high frequency touch surfaces at critical times when a person is ill and actively shedding pathogens, or is immune-compromised, or where a salmonella-contaminated chicken is placed onto a kitchen surface. 

Despite the fact that the quality of estimates generated by QMRA are dependent on the quality of the available data, the downsides are probably no more than those associated with intervention studies, where validity of the data is compromised by difficulties of experimental design, controlling variables, and costs of generating dose-response data.

## Keeping surfaces hygienically clean – how long is the safety target level sustained?

Hygienic cleaning of hands, fabrics and surfaces is at its most effective in reducing infection risks when used immediately before or after actions where there is identified risk of spread of pathogens, such as after preparation of raw foods, before changing a dressing or catheter, after visiting the toilet or after changing a baby’s nappy.

Where hygienic cleaning procedures are applied to hands, surfaces and fabrics as part of daily or weekly routines, there is constant risk of recontamination with pathogens as well as other microbes between treatments. In all settings, potentially pathogenic microbes are constantly shed or spread into the environment and onto environmental surfaces, from sources such as people (infected or colonized), domestic animals, contaminated air, food and water [18]. Understanding how bioburdens of pathogenic organisms accumulate, and fluctuate on surfaces after hygienic cleaning is required to determine how often surfaces, particularly frequent hand contact surfaces, should be hygienically cleaned. Studies from hospital and domestic settings suggest that ambient “total aerobic colony count” bioburdens return to levels which were present prior to applying a hygiene procedure within time periods of 1.5 to 2.5 h [34], [66]. Two studies [67], [68] show how MRSA rapidly recontaminates high-touch sites in Intensive Care Units after hygienic cleaning. The rate of recontamination with pathogens will depend on a number of factors. For example, risks from ambient spread of pathogens in the domestic environment are likely to be less than in healthcare settings, but even in domestic settings, will increase where a family member is infected with a respiratory virus or norovirus. Similarly, the risk for hospital patients is likely to increase where, a patient on the ward is actively shedding *S. aureus*. 

In order to make some assessment of the required frequency of cleaning, Bogusz et al. [69] studied the effect of detergent-based cleaning at near-patient sites (lockers, left and right bedrails and overbed tables) in 30 hospital bed spaces over 48 h. There was significant reduction in total aerobic colony counts (ACC) (360 sites) from 6.72 to 3.46 ACC/cm^2^ at 4 h after cleaning. Counts increased to 4.89 and 5.27 ACC/cm^2^ at 24 and 48 h respectively for all sites. Levels on bed rails and lockers, but not overbed tables, remained below 5 cfu/cm^2^ for 24 h after cleaning. Staphylococci (methicillin-susceptible and methicillin-resistant *S. aureus*) decreased 2–4 h after cleaning before increasing again, but failed to reach pre-clean levels. From this, the authors concluded that infection risks from near-patient sites could potentially be controlled by daily cleaning with single-use detergent wipes. 

Keeping hospitals and other settings visibly clean has long been regarded as an aesthetic necessity, but there is increasing evidence that this cleaning plays a part in managing infections, although most of this data comes from manufacturing environments [70] and studies associated with healthcare settings [1], [7], [58]. In healthcare settings, environmental surfaces are routinely cleaned and/or disinfected, according to predetermined policies (e.g., hourly, daily, twice weekly, etc.) or when surfaces appear visibly dirty. Frequently touched items (bed rails, locker tops etc. telephones, handles, taps, light switches, levers, knobs, buttons, keyboards, push plates) are cleaned more frequently than floors and furniture where risks of pathogens are less. In recent years microbiological standards have been proposed for hospital cleaning. As reviewed by Dancer [7], aerobic colony counts of <2.5 to 5 cfu per cm^2^ on hand touch sites and <1 cfu/cm^2^ hospital pathogen (e.g., MRSA, vancomycin-resistant enterococci, *C. difficile*, etc.) have been proposed and tested as microbiological benchmarks. The two benchmarks appear to be related, in that higher levels of aerobic colonies on hand touch sites are more likely to be associated with the presence of *S. aureus* and MRSA [71]. Similar counts for food preparation surfaces form the basis of the monitoring framework set up by the food industry [70]. These standards are not based on any quantitative assessment of clinical risk, but provide benchmarks for monitoring whether standards of cleaning are being maintained, and giving early warning of changes (cleaning quality and frequency, environmental changes etc.) which might increase the infection risk. 

It is recognized that, as sites can rapidly become contaminated after cleaning, surface coatings with prolonged microbiocidal activity might be a useful adjunct for controlling recontamination, particularly for high frequency touch surfaces in high risk settings such as healthcare settings. Bioactive surfaces include heavy metals such as copper, zinc, silver or titanium, or biocides. By replacing or coating frequent contact surfaces with these materials, it may be possible to delay recontamination in a more sustained manner than can be achieved with periodic cleaning and disinfection alone, and thereby further reduce infection risks. Muller et al. [72] conducted a systematic review of the use of antimicrobial surfaces in patient rooms. Eleven studies assessed the effect of copper (N=7), silver (N=1), metal-alloy (N=1), or organosilane-treated surfaces (N=2) on microbial contamination. Copper surfaces demonstrated a median (range) reduction of microbial contamination of <1 log LR (<1 to 2 LR). In addition, a study of copper surfaces and one of copper textiles demonstrated reduction in health care-associated infections, but the authors concluded that both studies were at high risk of bias.

A major obstacle to developing effective antimicrobial surfaces is the development of stage 3 test models. Standard test protocols include an International Standards Organisation (ISO) test [73] and an ASTM test [74]. Neither of these testing conditions would seem to reflect conditions found in practice, since they involve testing against liquid bacterial cultures. In a recent study Campos et al. [75] compared the efficacy in ASTM and ISO standards with that determined by a “dry droplet” method. The varying results between protocols led them to conclude that efficacy of antimicrobial surfaces cannot be easily and reproducibly compared.

## Conclusions – an integrated approach to optimizing hygiene practice

This review proposes a hygiene assurance framework for developing, standardizing and comparing efficacy of hygiene procedures. Importantly it represents a framework which can be used in any type of setting (healthcare, domestic, industrial etc.) and applied to any type of site or surface (hands, fabrics and environmental sites surfaces) in order to prevent the transmission of infection.

The key feature of this framework is the inclusion of stage 3 models within a 4 stage testing protocol. Where stage 1 and stage 2 models assess the performance of disinfectant products (proof of principle), stage 3 models assess the state of the surface being treated (i.e. its fitness for purpose) i.e. they assess the ability of the procedure to break the chain of infection transmission. This allows us to compare efficacy of hygiene procedures that rely solely on removal of microbes with those that also employ chemical or thermal inactivation. This makes it possible to ensure that a consistent “safety target level” is achieved regardless of the procedure used. It also ensures that new technologies are at least as effective as existing ones. 

Whereas stage 4 intervention studies are considered the gold standard for establishing the impact of hygiene procedures on infection rates, they have significant limitations. Not only are they costly to perform and difficult to standardize, they rely on compliance by users in terms of frequency of use and method of application. If a procedure fails to show clinical benefit in stage 4 studies, without stage 3 study data, it is unclear whether or to what extent this might be due to the inadequacy of the procedure. Stage 3 models enable development of hygiene procedures which are most likely to deliver maximum efficacy, before being tested through a stage 4 intervention or QMRA study. A procedure which fails to show “fitness for purpose” in stage 3 is unlikely to show clinical benefit.

Similar principles are now being advocated by McDonald and Arduino [76] for resolving disagreements about the infection control benefits of no touch disinfection strategies for terminal disinfection [77], [78], [79]. The authors argue that, since demonstrating the clinical impact of environmental cleaning and disinfection technologies remains so challenging, a stepwise evidentiary hierarchy should be adopted, which involves not only efficacy studies as required for EPA [80] but also studies using models simulating practical conditions to establish whether interventions have “fitness for purpose”. 

The downside of stage 3 models is that the closer and more relevant they are to practical use conditions, the more difficult it is to control test parameters in order to achieve repeatable and reproducible results [15]. This is particularly so for hygiene procedures where mechanical pressure etc. needs to be controlled. 

Adopting approaches based on hygiene assurance levels also has other potential advantages.

- Firstly it obviates the need for multiple clinical trials to demonstrate efficacy for every new cleaning or cleaning and disinfection procedure. In clinical practice, different drug treatments for a disease can have radically different efficacy, because of differences in mode of action on the human body. By contrast, exposure to a given residual dose of pathogens (e.g. from surface to hand to mouth) carries the same level of risk, regardless of the hygiene procedure (heat, disinfection, removal) which has been used to reduce the number of infectious particles. Carling and Huang [1] point out that, once a safety target level for a practice (e.g hand hygiene, food contact surface hygiene) is determined through intervention studies or QMRA, establishing that this is associated with acceptably low residual infection risk, it can be used as a stage 3 performance standard for any hygienic cleaning practice to be used in that situation.
- In public and domestic situations, there is pressure to deliver hygiene in a manner which is sustainable. Use of stage 3 models facilitates the development of procedures which maximize protection against infection whilst minimizing impact on the human and environmental microbiome, and ensuring prudent usage of antimicrobial agents, detergents, water and energy. Data from stage 1 and 2 with stage 3 testing facilitates an understanding as to how inactivation and removal processes can work synergistically to optimize LRs on hands, surfaces and fabrics. Together they can be used for developing new approaches to optimize hygienic cleaning, including new cleaning and disinfection agents, new technologies, and surface modification to facilitate detachment.
- Importantly, use of consistent hygiene assurance framework enables knowledge transfer and sharing between hygiene professionals in different settings, whilst the use of consistent terminology facilitates consistent communication with those (including the general public) charged with putting hygiene/infection control policy into practice.

If we are to restore understanding of hygiene, and confidence in its vital role in containing the burden of infectious disease, we must present healthcare professionals, policy makers, regulatory authorities and the public with a well argued, scientifically supportable approach. The hygiene assurance level approach described in this paper provides a rational framework for developing and comparing effective hygiene procedures. Although much further work is required to develop this framework, it provides a means to develop processes and products which work together to deliver maximum health benefit.

## Notes

### Competing interests

The authors declare that they have no competing interests.

## Figures and Tables

**Table 1:**
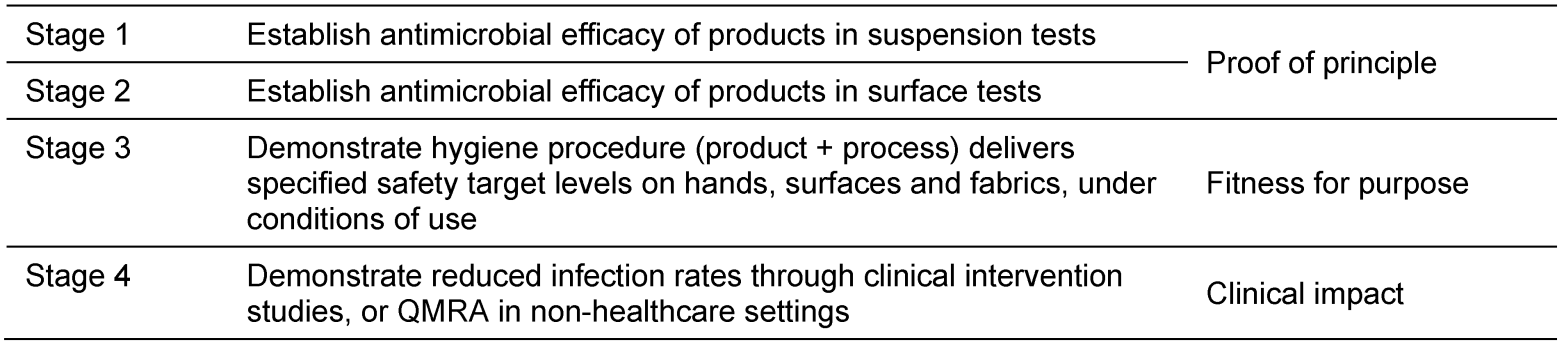
Proposed hierarchy of testing for hygiene procedures used on contaminated hands, environmental surfaces and fabrics

**Table 2:**
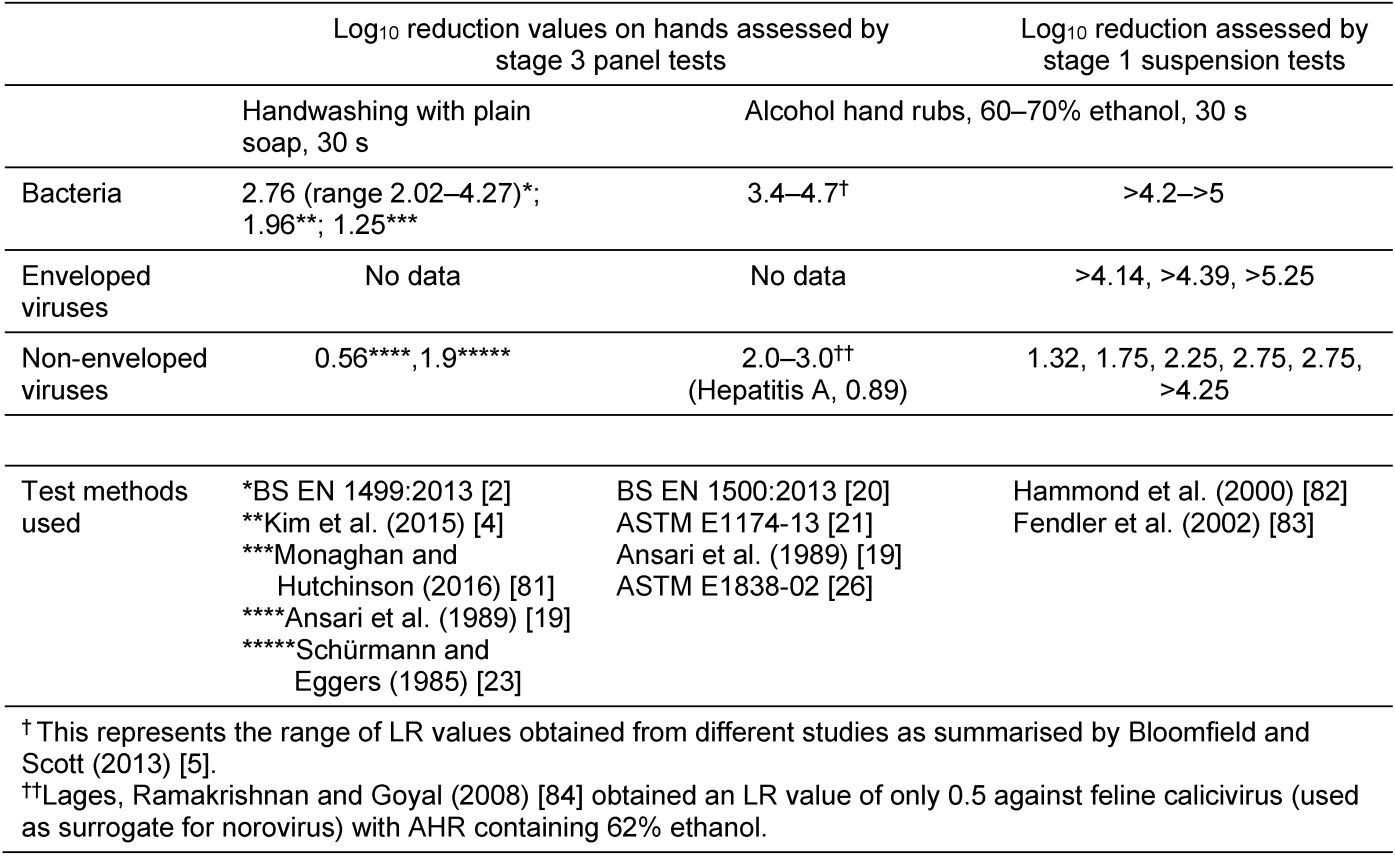
Comparison of the hygiene effectiveness of handwashing with soap and alcohol handrubs against bacteria and viruses using data obtained from published studies (in part from Bloomfield et al. (2007) [3]; Bloomfield and Scott (2013) [5])

**Table 3:**
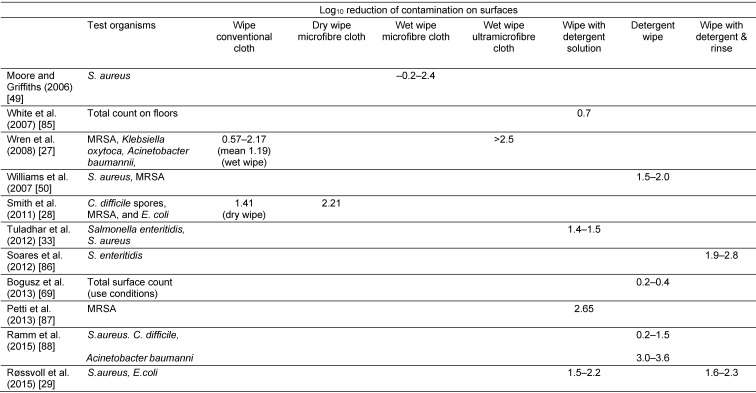
Estimations of Log_10_ reduction in bacterial contamination on surfaces achieved by dry and wet wiping, wiping with detergent, wiping with detergent plus rinsing, and using a detergent wipe
